# Effect of papaya (*Carica papaya*) seed as phytogenic feed additives on egg performance, egg quality and blood serum biochemical constituents of layer hens

**DOI:** 10.1002/vms3.1295

**Published:** 2023-10-17

**Authors:** Meshesha Dissa, Yonatan Kassu Yesuf, Ermias Belete

**Affiliations:** ^1^ Department of Animal Science Wolaita Sodo University Wolaita Sodo Ethiopia

**Keywords:** egg production, egg quality, layer chickens, papaya seed phytogenic feed additive, serum biochemical

## Abstract

**Background:**

Supplementing poultry diets with certain natural phytogenic additives has positive impacts on performance parameters like feed intake, egg production and quality of layer hens.

**Objectives:**

The study aimed to examine how supplementing papaya seed as phytogenic feed additive (PFA) to the diets of layer chickens affected their egg production, quality and blood serum biochemical parameters.

**Methods:**

One hundred twenty 28‐week‐old Bovans brown laying hens were randomly assigned to one of four treatments with varying levels of papaya seed supplementation at 0 g/kg (T1), 5 g/kg (T2), 10 g/kg (T3) and 15 g/kg (T4) in a complete randomized design.

**Result:**

Layer chickens supplemented with 0.5% (T2), 1% (T3) and 1.5% (T4) papaya seed as a PFA had significantly (*p* < 0.05) improved dry matter intake, egg production%, egg mass and feed conversion. On the other hand, papaya seed supplementation decreased (*p* < 0.05) the bodyweight gain of laying hens. Papaya seed supplementation significantly (*p* < 0.05) increased egg weight, length, width, yolk height and yolk colour compared to the control group. Serum total protein on T4 and liver enzyme of alkaline phosphatase on T3 were significantly greater (*p* < 0.05) than in the control group. However, there were no significant differences (*p* > 0.05) in serum albumin, glucose, total cholesterol, triglyceride and aspartate aminotransferase levels among treatments laying hens.

**Conclusion:**

Papaya seed can be a potential candidate as a PFA at 5–15 g/kg of the basal diet to improve egg‐laying performance and quality without deteriorating effect.

## Graphical abstract

- Supplementing layer hen diets with 5–15 g/kg dried papaya seed increased egg production by 5%, egg mass, feed efficiency, egg weight, yolk height and serum protein levels in laying hens.
- These results demonstrate papaya seed's potential as a natural feed additive to improve egg yield, quality and maintain the liver function by reducing liver inflammation in layer hens.

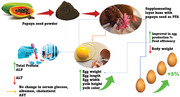

## 1 INTRODUCTION

Phytogenic feed additives (PFA) derived from plants have shown benefits as alternatives to antibiotics and synthetic supplements in poultry production, improving feed efficiency, growth, egg production and overall health (Khan et al., 2019).

Several studies have shown that adding certain natural supplements PFA to a chicken's diet have a positive impact on their egg production, weight, quality and overall health. For example, researchers found that including fennel seed, black cumin seed and hot red pepper at a ratio of 0.5% of the basal diet significantly increased egg production, egg weight (EW), egg mass and feed conversion ratio (FCR) when compared to a non‐supplemented group (Abou‐Elkhair et al., 2018). Additionally, Abd El‐Hack and Alagawany (2015) found that adding rosemary leaf at a rate of 3 g/kg led to an increase in egg production and egg mass. Moreover, thyme and oregano essential oils have improved feed efficiency, egg mass and egg production at supplementation levels around 1%–2%. Furthermore, garlic powder has been shown to improve EW and shell quality (Chowdhury et al., 2018). Ginger extract supplemented at 200–400 mg/kg resulted in better feed utilization and internal egg quality measures like Haugh units (Hus) and yolk weight in layers (Herkeľ et al., 2018).

Papaya seed is one prospective natural supplement for poultry, as papaya is a common tropical fruit cultivated worldwide and the seeds are often discarded as an agricultural by‐product. Papaya peels and seeds are generated in large amounts each year, especially in Ethiopia (7772.77 tons), and the seeds make up roughly 14.3% of the overall fruit (Li et al., 2015; Saleh et al., 2019). In papaya seed, there are significant amounts of crude protein, essential amino acids, fatty acids, crude fibre and minerals like calcium, phosphorus, magnesium, iron and salt (Beski et al., 2015). In addition, there are vitamins C and E (Muazu & Aliyu‐Paiko, 2020). Furthermore, papaya seeds contain beneficial bioactive like carotenoids, phenolic acids, benzyl isothiocyanates, carpaine, caricine, papaya oil and glucotropaeolin (Maisarah et al., 2014; Salla et al., 2016).

However, limited research has explored the direct impacts of supplemented papaya seeds on productivity metrics like egg yield and quality in laying hens. Earlier studies found 0.5%–2% papaya seed powder reduced parasite loads and increased feed intake in pullets but did not assess egg performance (Soedji et al., 2017). Additionally, papaya pomace supplementation did not influence feed intake in layers, though egg cholesterol content decreased (Rahmasari et al., 2022).

Therefore, this study aims to evaluate the effect of papaya seed as feed additives on egg production, quality and blood serum biochemical parameters of laying hens. The results will elucidate the potential for discarded papaya seeds to improve productivity in layers as an inexpensive, natural supplement.

## 2 MATERIALS AND METHODS

### 2.1 Preparation of papaya seed additive

A ripened papaya (*Carica papaya)* seed was collected from local juice shops and all unnecessary materials (peels, pulps and dirt) were removed by hand‐picking from the seeds. After removing coarse peels and pulps, the seeds were washed with tap water to remove small pulps and slimy substances. The washed seeds were soaked in a 3% hydrogen‐peroxide (H_2_O_2_) solution (1‐L tap water with 3 mL of H_2_O_2_ ratio) for 30 min to clean the fungus and any contaminants (Sanna et al., 2022). The soaked seeds were filtered after 30 min, air‐dried for eight consecutive days and stored at the temperature of 12–140°C. Dried papaya seeds were coarsely ground using a mortar and pestle and sieved through a 5 mm mesh screen for adding PFAs in the layer rations.

### 2.2 Experimental diets

The conventional mash diet for laying chickens was based on corn and soybean meal. The grounded meals were weighed to formulate a quintal of layers of mash, as indicated in Table 2. Only a basal diet was offered to the control group T1 (without papaya seed PFAs) until the end of the experimental period. The treatment groups were fed the same amount of basal diet with air‐dried papaya) seed at 5 g/kg (T2), 10 g/kg (T3) and 15 g/kg (T4). Proximate analysis was carried out to estimate the experimental diets according to the standard procedure of Association of Official Analytical Chemists AOAC (2005). The experimental diets were formulated to meet the nutrient requirements for layer hens according to the recommendations of the commercial layer (Bovans brown lite layers) commercial management guide. Table 1 presents the proximate chemical composition of the papaya seed.

**TABLE 1:** Proximate analysis of papaya seed diet.

Feed additive	Chemical composition (%)				
Papaya seed	DM	CP	CF	EE	Ash
	92.41	38.38	14.56	26.19	6.63

Abbreviations: CF, crude fibre; CP, crude protein; DM, dry matter; EE, ether extract.

**TABLE 2:** Ingredients and nutrient composition of the diet.

Ingredients	Amount
Corn	54.50
Wheat middling	10.36
Soybean meal (44% CP)	20.29
Niger seed cake	8.00
Limestone	5.80
Lysine	0.07
Methionine	0.14
Vit‐min premix^a^	0.5
Salt	0.34

Analyzed chemical composition	
Dry matter	91.10
Metabolizable energy (kcal/kg)	2755.2
Crude protein	16.45
Crude fat	5.60
Crude fibre	7.2
Available calcium	3.84
Available phosphorous	0.35
Ash	11.53

Abbreviation: CP, crude protein.

^a^ Supplied per kg of diet: vitamin A 12,000 IU, vitamin D3 3000 IU, vitamin E 40 mg, vitamin K3 3 mg, vitamin B1 2 mg, vitamin B2 6 mg, vitamin B6 5 mg, vitamin, B12 0.02 mg, niacin 45 mg, biotin 0.075 mg, folic acid 2 mg, pantothenic acid 12 mg, manganese 100 mg, zinc 600 mg, iron 30 mg, copper 10 mg, iodine 1 mg, selenium 0.2 mg and cobalt 0.1 mg.

### 2.3 Experimental design and management of layer chickens

Bovan brown layer chickens at 28‐week‐old with similar body weight were obtained from a private farm. Individual laying hens were weighed and randomly assigned in a complete randomized design into four dietary treatment groups (T1): basal diet + 0 g/kg papaya seed; (T2): basal diet + 5 g/kg papaya seed; (T3): basal diet + 10 g/kg papaya seed; (T4): basal diet + 15 g/kg papaya seed. Each treatment has four replicates consisting of 10 layers each. The experiment was conducted between 28 and 52 weeks of age (24 weeks experimental period).

The cage was designed at 1.8 × 1 × 0.5 m^3^ for each replicate with feed and water trough. Clean water was provided ad libitum during the experimental period. The layers were vaccinated against common poultry diseases (fowl typhoid at 32 weeks of age). The lighting program was 16 h of continuous light per day. The layers were kept in optimal, standard bioclimatic and welfare conditions and managed according to the experimental animal protocol approved by the ethical committee and institutional review board (IRB) (reference number WSU/41/14/1023).

### 2.4 Data collection

#### 2.4.1 DM intake and egg production performance

The dry matter intake (DMI) was determined by the difference between daily feed offered and daily feed refused at the dry matter base. The body weight gain of layers was calculated as the difference between the final and initial body weight to observe the body weight change of layers. The FCR was expressed as grams of feed consumed per grams of egg produced, and layers efficiently convert the feed into an egg (Abou‐Elkhair et al., 2018).

The egg production performances were expressed as the percentage of each replicate's hen‐day and hen‐housed egg production %. The egg mass was computed as described by North (1984) by multiplying the average EW in grams with the hen‐day egg production for each pen.

#### 2.4.2 External and internal egg quality

For the measurement of external and internal egg quality, four eggs per replicate were randomly collected at 30, 40 and 50 weeks of age to assess external and internal egg quality traits’ eggs were weighed each time using a sensitive balance with a precision of 0.01 g. Digital caliper measured egg length from the tip of the narrow (sharp) end to the broad end. A digital caliper measured the width of each egg at the centre, and the egg shape index was computed by dividing egg width by egg length × 100 (Doyon et al., 1986). The eggshell weight (g) was measured by electronic balance by removing the shell membrane. The shell thickness (mm) was calculated from the narrow (sharp end), equator and broad end (air cell) of the shell by using a digital caliper by removing the shell membrane. The average of three sites was considered eggshell thickness (Hosseinzadeh et al., 2010).

Each egg was carefully opened on a flat glass plate and kept for 3 min for internal egg quality analysis. A gauge micrometre measured individual egg yolk height (calibrated in mm). Yolk diameters were measured using a digital caliper. The colour of the yolk was determined by the Roche colour fan from 1 to 15 colour (Hoffman‐La Roche Ltd.). After separating the yolk from the albumen, the yolk weight was measured using an electronic balance. The yolk index was calculated as yolk height divided by the yolk diameter × 100 (Doyon et al., 1986). Albumen height (AH) was measured by gauge micrometre at two sides, the average was considered AH, and the weight of albumen was measured by sensitive balance. HUs were calculated from the records of AH and EW using the HU formula: HU = 100 × (log AH + 7.57 − 1.7EW^0.37^) (Haugh, 1937).

### 2.5 Blood serum preparation and biochemical constituents

At the end of the experiment, blood samples were obtained from 24 layers. Two layers per replication were randomly selected, and 3 mL of blood per bird was drawn from the brachial vein using a 5 mL sterile disposable syringe and transferred into a sterilized tube. The blood serum was extracted from the tubes using a centrifuge and kept at −20°C after being spun for 5 min (Kilinc & Karaoglu, 2020). Serum blood biochemical constituents were determined by the chemistry auto analyser COBAS 6000. Moreover, measurements of the liver's working enzymes aspartate aminotransferase (AST), alanine aminotransferase (ALT) and alkaline phosphatase (ALP) were performed. Commercially available kits (RANDOX) were used to analyse the serum for total protein, albumin, glucose and total cholesterol in the enzymatic endpoint method triglycerides in the GPO–PAP method.

### 2.6 Statistical analysis

The collected data were subjected to a one‐way analysis of variance using SAS statistical package version 9.4. Duncan Multiple Range test evaluated treatment differences between group means at a 5% significance level.

## 3 RESULTS

### 3.1 Effects of papaya seed feed additive on egg production and FCR

The effect of dietary supplementation or additives of papaya seed on DMI, body weight gain, egg production, egg mass and FCR of the layer is presented in Table 3. Papaya seed additives significantly (*p* < 0.05) increased daily DMI, egg production, egg mass and FCR of the layer hen. The daily DMI was increased (*p* < 0.05) as the papaya seed additives increased. Papaya seed as a PFA at 5, 10 and 15 g/kg significantly (*p* < 0.05) improved egg production % by 3.27, 3.11 and 4.98, respectively. FCR was improved by 1.97%, 2.96% and 3.45% at the levels of the PFA increased from T2–T4 groups, respectively. The layer body weight change was significantly (*p* < 0.05) different among treatments. Papaya seed slightly (*p* < 0.05) decreased the body weight gain of layer chickens compared to the control group. However, there was no difference (*p* > 0.05) in body weight among papaya seed PFAs groups.

**TABLE 3:** Effect of papaya seed feed additive on the performance of layer chickens.

Parameters	Dietary treatment				*p*‐Value
	T1	T2	T3	T4	
DMI (g/day/hen)	116.44 ± 0.09^d^	117.52 ± 0.05^c^	118.34 ± 0.02^b^	118.63 ± 0.11^a^	<0.001
IBW (kg)	1.54 ± 4.16	1.53 ± 5.77	1.54 ± 7.02	1.54 ± 6.21	0.8912
FBW (kg)	1.85 ± 3.15^a^	1.82 ± 5.17^b^	1.83 ± 2.11^b^	1.82 ± 2.67^b^	0.0008
BWC (g)	310.76 ± 2.3^a^	287.23 ± 6.51^b^	289.47 ± 3.71^b^	279.85 ± 5.87^b^	0.011
HDEP (%)	81.69 ± 0.17^c^	84.36 ± 0.10^b^	84.23 ± 0.30^b^	85.76 ± 0.40^a^	<0.001
HHEP (%)	81.69 ± 0.17^c^	84.36 ± 0.10^b^	84.23 ± 0.30^b^	85.76 ± 0.40^a^	<0.001
Egg mass	46.67 ± 0.13^d^	49.84 ± 0.06^c^	50.66 ± 0.18^b^	51.94 ± 0.25^a^	<0.001
FCR	2.03 ± 0.053^a^	1.99 ± 0.031^b^	1.97 ± 0.062^c^	1.96 ± 0.041^c^	<0.001

*Note*: Mean values within the same row having different letters (a, b and c) are significantly different at (*p* < 0.05); T1: basal diet + 0 g/kg papaya seed; T2: basal diet + 5 g/kg papaya seed; T3: basal diet + 10 g/kg papaya seed; T4: basal diet + 15 g/kg papaya seed.

Abbreviations: BWC, body weight change; DMI, dry matter intake; FBW, final body weight; FCR, feed conversion ratio; HDEP, hen day egg production; HHEP, hen housed egg production; IBW, initial body weight; *p*‐Value, probability.

### 3.2 Effects of papaya seed feed additive on egg quality

Table 4 shows the effect of papaya seed as a PFA on the quality of layers’ eggs. EW increased by 2.1, 2.9 and 3.4 g in the T2, T3 and T4 groups, respectively (*p* < 0.05). In addition, the length, width, yolk height and colour of T2, T3 and T4 groups eggs have all been increased (*p* < 0.05). The albumen weight was not significantly (*p* > 0.05) increased in the T2 and T4 groups. However, in treatments T3 and T4 group, the yolk weight, index, as well as albumen weight, were significantly (*p* < 0.05) increased. However, there is no significant (*p* > 0.05) difference in yolk index. The addition of papaya seed feed additive, on the other hand, had no effect (*p* > 0.05) on yolk diameter, HU, AH or shell weight of chicken eggs.

**TABLE 4:** The effect of papaya seed feed additive on egg quality of layers chickens.

	Dietary treatment				
Parameters	T1	T2	T3	T4	*p*‐Value
Egg weight (g)	58.53 ± 0.42^b^	60.63 ± 0.49^a^	61.45 ± 0.37^a^	61.90 ± 0.63^a^	<0.001
Egg length (mm)	54.25 ± 0.33^b^	56.67 ± 0.47^a^	55.92 ± 0.31^a^	55.50 ± 0.50^a^	0.0005
Egg width (mm)	43.83 ± 0.39^b^	45.75 ± 0.41^a^	45.25 ± 0.37^a^	45.75 ± 0.33^a^	0.0016
Egg shape index (%)	80.95 ± 0.53	80.57 ± 0.59	80.93 ± 0.63	81.10 ± 0.89	0.9511
Shell weight (g)	6.52 ± 0.21	6.54 ± 0.14	6.54 ± 0.28	6.72 ± 0.14	0.8895
Shell thickness (mm)	0.33 ± 0.01	0.30 ± 0.01	0.30 ± 0.01	0.31 ± 0.01	0.1277
Yolk height (mm)	17.83 ± 0.42^b^	19.50 ± 0.36^a^	19.08 ± 0.26^a^	19.50 ± 0.34^a^	0.0041
Yolk diameter (mm)	39.67 ± 0.88	38.00 ± 0.35	38.00 ± 0.35	39.12 ± 0.47	0.0863
Yolk Index (%)	44.20 ± 1.03^b^	46.62 ± 1.12^ab^	48.17 ± 0.71^a^	49.01 ± 1.07^a^	0.0072
Yolk colour	7.92 ± 0.36^c^	8.83 ± 0.35^b^	9.08 ± 0.26^ab^	9.83 ± 0.32^a^	0.0016
Yolk weight (g)	17.14 ± 0.29^c^	18.43 ± 0.38^ab^	17.78 ± 0.39^bc^	18.79 ± 0.40^a^	0.0063
Albumen height (mm)	8.42 ± 0.19	8.33 ± 0.14	8.25 ± 0.13	8.25 ± 0.13	0.8408
Albumen weight (g)	34.79 ± 0.34^b^	35.50 ± 0.99^ab^	37.00 ± 0.37^a^	36.12 ± 0.44^ab^	0.0453
Haugh unit	91.92 ± 0.92	90.99 ± 0.75	90.35 ± 0.72	90.23 ± 0.69	0.4025

*Note*: Mean values within the same row having different letters (a, b and c) are significantly different at (*p* < 0.05); T1: basal diet + 0 g/kg papaya seed; T2: basal diet + 5 g/kg papaya seed; T3: basal diet + 10 g/kg papaya seed; T4: basal diet + 15 g/kg papaya seed.

Abbreviation: *p*‐Value: probability.

### 3.3 Effect of papaya seed feed additive on serum biochemical constitutes

The effect of papaya seed as a PFA on layers of blood serum biochemical constituents is presented in Table 5. The PFA of papaya seed at 15 g/kg (T4) significantly (*p* < 0.05) raised blood serum total protein. However, there was no significant (*p* > 0.05) difference in serum albumin, glucose, total cholesterol, triglyceride and AST liver enzyme compared to the control group. Papaya seed PFA significantly (*p* < 0.05) decreased ALT in T2, T3 and T4, respectively, compared to the control group. Papaya seed supplementation substantially (*p* < 0.05) decreased ALP in T2, whereas it increased in T3 group.

**TABLE 5:** Effect of papaya seed feed additives on biochemical blood constitutes of layer chickens.

	Dietary treatment				
Parameters	T1	T2	T3	T4	*p*‐Value
T Protein (g/dL)	4.00 ± 0.01^b^	4.32 ± 0.19^ab^	4.04 ± 0.08^b^	4.53 ± 0.10^a^	0.0346
Albumin (g/dL)	1.82 ± 0.02	1.84 ± 0.08	1.82 ± 0.04	1.95 ± 0.04	0.2753
Glucose (mg/dL)	196.57 ± 12.15	185.77 ± 4.64	177.00 ± 28.67	170.87 ± 16.25	0.0686
TCH (mg/dL)	137.27 ± 8.14	141.23 ± 2.57	146.93 ± 2.00	150.00 ± 1.87	0.2602
TRIG (mg/dL)	96.48 ± 7.39	97.0.9 ± 12.13	98.2.1 ± 7.70	98.790 ± 6.66	0.5278
ALT (U/L)	11.63 ± 1.24^b^	8.13 ± 0.37^c^	8.00 ± 0.45^c^	10.40 ± 1.60^a^	0.0017
AST (U/L)	153.00 ± 4.76	143.27 ± 11.82	143.50 ± 6.23	135.50 ± 7.52	0.5297
ALP (U/L)	545.67 ± 9.82^b^	268.3 ± 17.03^c^	760.67 ± 13.69^a^	563.00 ± 10.41^b^	<0.001

*Note*: Mean values within the same row having different letters (a, b and c) are significantly different at (*p* < 0.05); T1: basal diet + 0 g/kg papaya seed; T2: basal diet + 5 g/kg papaya seed; T3: basal diet + 10 g/kg papaya seed; T4: basal diet + 15 g/kg papaya seed.

Abbreviations: ALP, alkaline phosphatase; ALT, alanine transferase; AST, aspartate aminotransferase; g/dl, gram per decilitre; *p*‐Value, probability; TCH, total cholesterol; TRIG, triglyceride; U/L, unit per litre.

## 4 DISCUSSION

During the experimental period, supplementation of papaya seeds as feed additive increased DMI linearly. Among the supplemented groups, the highest daily DMI was observed on T4 group (15 g/kg of PFA of papaya seed). The current result agrees with that of Soedji et al. (2017), who supplemented papaya seed to ISA Brown male (layer‐type) chicks at 0.5%, 1% and 2% of basal diet and observed an increased feed intake. This finding disagrees with Tamiru et al. (2021), who reported that 20–32 weeks‐age layer chickens fed papaya pomace (seeds, peels and pulps) at 2.5%, 5% and 7.5% inclusion levels showed no effect on feed DM intake. According to Salla et al. (2016), the active ingredients in papaya seeds, such as alkaloids, flavonoids, phenols and amino acids, can stimulate saliva and bile secretions, enhance the activities of digestive enzymes like proteolytic enzyme and increase the palatability of the basal diet. These factors could be possibly attributed to the fact that responsible for improving the DMI of papaya seed PFA groups.

In the current study, the decrease in body weight gain of layer chickens supplemented with papaya seed at 5, 10 and 15 g/kg, maybe layers utilized the feed they consumed to produce more eggs than the control group. This result aligned with previous finding of Mabusela et al. (2018), who reported that supplementation levels of 1%, 3% and 5%, *Moringa oleifera* seed to layer diet, lower the body weight gain of layer chickens.

The improvement observed in the present study may be an effect of the active compounds of papaya (benzyl isothiocyanate and papain), which had positive effects on the destruction or lowering of parasites, as a result, ample availability of nutrients for absorption and egg production. Additionally, papaya seed has high in nutritional value, like protein, amino acids and fatty acids, which may improve the egg production performance of the chickens. The current result agrees with Kaya et al. (2014), who reported that supplementing grape seed to 44–56‐week‐aged Lohman layer chickens at 0.5%, 1% and 1.5% increased egg production by 13.88%, 12.84% and 18.03%, respectively.

The current study observed the highest hen day and hen‐housed egg production % was obtained from laying hens supplemented with 15 g/kg of PFA of papaya seed. This might be because the increasing level of papaya seed also increased the level of active compounds like the flavonoids in the diet and positively enhanced egg production by facilitating digestion and nutrient absorption. Novitasari et al. (2018) and Leke et al. (2018) revealed that the flavonoid components (myricetin and kaempferol) contained in natural plant feed additives had an estrogenic activity‐inducing effect through regulating the hypothalamic gonadal axis of laying chicken. Previous studies employing papaya pomace, flaxseeds and fenugreek seeds for layers have confirmed this hypothesis (Saleh et al., 2019; Tamiru et al., 2021). The highest egg mass was recorded in the PFA of papaya seed at 15 g/kg group hens compared with 5 and 10 g/kg group hens. The current result agrees with El‐Saadany et al. (2022), who revealed that the PFAs of pumpkin seed products in 24–32 weeks age laying hens improved egg mass. Since, both pumpkin and papaya seeds contain high levels of protein, essential amino acids, unsaturated fatty acids and fibre, which can provide added nutritional value to poultry diets (Nakasone & Paull, 1998; Nkukwana et al., 2014).

Similarly, Abou‐Elkhair et al. (2018) reported that Lohmann brown 32–40 weeks aged laying chickens supplemented with 0.5% fennel seed and black cumin seed increased egg mass. The increase in egg mass in the current study is due to the increased daily egg production % and EW. In the current study, better FCR has been observed in PFA of papaya seed treatments is also, due to the positive effect of papaya seed on egg‐laying performance and EW.

Layers fed with diets supplemented papaya seed increased EW, length, width, yolk height and colour compared to the control group. Leke et al. (2018) reported that papaya seed as organic feed by 2.5% inclusion level increased the egg quality of non‐commercial layer chickens. The improvement in EW of the present study may be because papaya seed additives increased the daily DMI, which might also increase the intake of nutrients. Furthermore, papaya seeds contain different amino acids like lysine, methionine and fatty acids, particularly linoleic acid (Alashi et al., 2013), which may increase EW. In the current study, supplementation of papaya seed did not affect egg shape index, eggshell weight, eggshell thickness, AH and HU. The data of the current study agree with that of Rahmasari et al. (2022), who declared that 0.3%, 0.6% and 1.2% of papaya seed inclusion in 75–83‐day‐old layer quail diet indicated no change in eggshell thickness. Also, El‐Saadany et al. (2022) revealed that the supplementation of pumpkin seed product to 24–32 weeks age layer chicken showed no difference in eggshell thickness. The improvement in yolk colour could be due to carotenoids such as β‐cryptoxanthin, lutein and β‐carotene in papaya pomace (Aroche et al., 2018). Similar to the findings of the present study, Tamiru et al. (2021) and Leke et al. (2018) reported that yolk colour intensity was significantly improved when papaya pomace and peel were fed to layer chickens.

Supplementation of papaya seed additive at 15 g/kg (T4) raised the blood serum total protein. The finding showed that total serum protein was at average reference ranges: 4.55–6.46 g/dL of chicken (Udoyong et al., 2010). According to Guo et al. (2022), the dietary supplementation of 100 mg/kg Masson pine (*Pinus massoniana*) to layer diet increased the total serum protein. Also, Yonatan et al. (2017) reported that black cumin and fenugreek at 1% and 2% in broiler chicken diets increased serum protein. However, the current finding is not in agreement with that of Muazu and Aliyu‐Paiko (2020), who reported that supplementation of papaya seed up to 1% to broiler chickens showed no change in serum total protein. The increase in serum total protein observed in the current study may be due to the increased availability of dietary protein, which was achieved by supplementing papaya seed PFAs at 15 g/kg. However, papaya seed does not contain a hepato‐protein inhibitor (Eid et al., 2021), which can lead to an accumulation of protein in the blood. Therefore, the absence of a hepato‐protein inhibitor in papaya seed may have also increased serum total protein levels. The total albumin, glucose, cholesterol and triglycerides were not changed due to the PFAs of papaya seed to layer chicken and the serum total albumin were within the standard reference range 1.17–2.74 g/dL (Meluzzi et al., 1992); serum glucose 130–270 mg/dL and total cholesterol: 125–200 mg/dL (Reece et al., 2015). A similar result was reported by Gozde and Mevlut (2020), who noted that laying hens supplemented with 100, 200 and 300 mg/kg of grape seed oil and *Hypericum perforatum* to layer diet showed no change in total cholesterol and triglyceride.

Supplementation of papaya seed as a PFA decreased the liver enzyme ALT in T2 and T3. This might be due to the presence of antioxidants (Vitamin C, Vitamin E and β‐carotene) and phenolic components in papaya seeds that may also sustain layer chicken liver function by lowering inflammatory liver enzymes. The decreases in AST and ALP activities support this interpretation, as their primary function is to channel amino acids into the citric acid cycle (Huang et al., 2006). The current finding disagrees with that of Kaya et al. (2014), who reported that supplementation of grape seed to 44–56‐week‐aged Lohman layer chickens at 0.5%, 1% and 1.5% did not affect serum ALT levels. According to Bolu et al. (2006), the ALT values become high when toxic substances cause liver damage. There is no particular change in AST concentration level among treatment of layer chickens. This may be because papaya seed supplementation does not negatively affect liver AST enzyme, and the finding at standard reference ranges for chicken is 70–220 U/L (Meluzzi et al., 1992). A similar result was reported by Gozde and Mevlut (2020), who noted that laying hens supplemented with *H. perforatum* to layer diet did not affect the liver enzyme of AST.

## 5 CONCLUSION

The dietary supplementation of papaya seed at 5, 10 and 15 g/kg to layer hens improved DMI, egg production, egg mass and FCR. Papaya seed supplementation positively affects some external and internal egg qualities. It increases serum total protein at a 15 g/kg supplementation level and maintains the liver function of layer chickens by reducing liver inflammation.

## AUTHOR CONTRIBUTIONS

*Conceptualization; methodology; data collection and writing – original draft*: Meshesha Dissa. *Conceptualization; process; analysis; supervision and manuscript writing and editing*: Yonatan Kassu Yesuf. *Conceptualization; data collection; supervision and writing and editing*: Ermias Belete.

## CONFLICT OF INTEREST STATEMENT

The authors declare no conflicts of interest.

## FUNDING INFORMATION

The author(s) reported there is no funding associated with the work featured in this article.

## ETHICS STATEMENT

This experiment followed the established animal experimental use, and care procedures of the Wolaita Sodo University Ethics Commission and the institutional review board (IRB) (reference number WSU/41/14/1023) employed.

### PEER REVIEW

The peer review history for this article is available at https://publons.com/publon/10.1002/vms3.1295.

## Data Availability

The data that support the findings of this study are available upon request.

## References

[vms31295-bib-0001] Abd El‐Hack, M. E. , & Alagawany, M. (2015). Performance, egg quality, blood profile, immune function, and antioxidant enzyme activities in laying hens fed diets with thyme powder. Journal of Animal and Feed Sciences, 24(2), 127–133.

[vms31295-bib-0002] Abou‐Elkhair, R. , Selim, S. , & Hussein, E. (2018). Effect of supplementing layer hen diet with phytogenic feed additives on laying performance, egg quality, egg lipid peroxidation and blood biochemical constituents. Animal Nutrition, 4(4), 394–400. 30564759 10.1016/j.aninu.2018.05.009 PMC6286622

[vms31295-bib-0003] Alashi, A. M. , Blanchard, C. L. , Mailer, R. J. , & Agboola, S. O. (2013). Technological and bioactive functionalities of canola meal proteins and hydrolysates. Food Reviews International, 29(3), 231–260.

[vms31295-bib-0004] Aroche, R. , Martínez, Y. , Ruan, Z. , Guan, G. , Waititu, S. , Nyachoti, C. M. , Más, D. , & Lan, S. (2018). Dietary inclusion of a mixed powder of medicinal plant leaves enhances the feed efficiency and immune function in broiler chickens. Journal of Chemistry, 2018, 1–6. 10.1155/2018/4073068

[vms31295-bib-0005] AOAC . (2005). Association of Official Analytical Chemist, Official Methods of Analysis (18th ed.) AOAC International, Suite 500, 481 North Frederick Avenue, Gaithersburg, Maryland 20877‐2417, USA.

[vms31295-bib-0006] Beski, S. S. M. , Swick, R. A. , & Iji, P. A. (2015). Specialized protein products in broiler chicken nutrition. A Review Animal Nutrition, 1(2), 47–53. 29766993 10.1016/j.aninu.2015.05.005 PMC5884466

[vms31295-bib-0007] Bolu, S. , Aklilu, H. , & Balogun, O. (2006). Effects of improved locally produced natural vitamin premix on the histology and specific enzyme activities of broilers. Indian Journal of Poultry Science, 6, 223–228.

[vms31295-bib-0008] Chowdhury, S. R. , Sarker, M. R. , Islam, K. S. , & Khan, M. J. (2018). Phytogenic feed additives as an alternative to antibiotics in poultry diet. Asian‐Australasian Journal of Animal Sciences, 31(7), 1065–1075.

[vms31295-bib-0009] Doyon, G. , Bernier‐Cardou, M. , Hamilton, R. M. G. , Castalgne, F. , & Randall, C. J. (1986). Egg quality 2. Albumen quality of eggs from five commercial strains of white leghorn hens during one year of lay. Poultry Science, 65, 63–66.

[vms31295-bib-0010] Eid, Y. Z. , Amber, K. A. , Hassan, M. S. , Hassan, R. A. , & Abo‐ouf, A. M. (2021). The protective role of silymarin to ameliorate the adverse effects of ochratoxin‐A in laying hens on productive performance, blood biochemistry, hematological and antioxidants status. Brazilian Journal of Poultry Science, 24(2), 1–8.

[vms31295-bib-0011] El‐Saadany, A. S. , El‐Barbary, A. M. , Shreif, E. Y. , Elkomy, A. , Khalifah, A. M. , & El‐Sabrout, K. (2022). Pumpkin and garden cress seed oils as feed additives to improve the physiological and productive traits of laying hens. Italian Journal of Animal Sciences, 21(1), 1047–1057.

[vms31295-bib-0012] Gozde, K. , & Mevlut, K. (2020). Effects of grape (*Vitis vinifera* L.) seed oil and St John's wort (*Hypericum perforatum* L.) extract supplementation into laying hens diets on performance, egg quality, and some blood parameters. International Journal of Science Letters, 2(1), 26–38.

[vms31295-bib-0013] Guo, Y. , Huang, S. , Zhao, L. , Zhang, J. , Ji, C. , & Ma, Q. (2022). Pine (*Pinus massoniana* Lamb.) Needle extract supplementation improves performance, egg quality, serum parameters, and the gut microbiome in laying hens. Frontiers in Nutrition, 9, 810462. 35223952 10.3389/fnut.2022.810462 PMC8868045

[vms31295-bib-0014] Haugh, R. R. (1937). The Haugh unit for measuring egg quality. The U.S. Egg and Poultry Magazine, 43, 552–555.

[vms31295-bib-0015] Herkeľ, R. , Gálik, B. , Bíro, D. , Šándor, K. , Fébel, H. , Pál, L. , & Zándoki, E. (2018). Effect of oregano and vitamin E dietary supplementation on the egg production and egg trait parameters of layer hens. Italian Journal of Animal Sciences, 17, 848–855.

[vms31295-bib-0016] Hosseinzadeh, M. , Ebrahimnezhad, Y. , Janmohammadi, H. , Ahmadzadeh, A. , & Sarikhan, M. (2010). Poultry byproduct meal: Influence on performance and egg quality traits of layers. International Journal of Agriculture And Biology, 12(4), 547–550.

[vms31295-bib-0017] Huang, X.‐J. , Choi, Y.‐K. , Im, H.‐S. , Yarimaga, O. , Yoon, E. , & Kim, H.‐S. (2006). Aspartate aminotransferase (AST/GOT) and alanine aminotransferase (ALT/GPT) detection techniques. Sensors, 6(7), 756–782. 10.3390/s6070756

[vms31295-bib-0018] Kaya, A. , Yıldırım, B. , Kaya, H. , Gül, M. , & Çelebi, Ş. (2014). The effects of diets supplemented with crushed and extracted grape seed on performance, egg quality parameters, yolk peroxidation and serum traits in laying hens. European Poultry Science, 78, 1–10.

[vms31295-bib-0019] Khan, S. H. , Iqbal, J. , & Javaid, A. (2019). Phytogenic feed additives in poultry nutrition: A review. Pakistan Veterinary Journal, 39(2), 153–164.

[vms31295-bib-0020] Kilinc, G. , & Karaoglu, M. (2020). Effects of grape (*Vitis vinifera* L.) seed oil and St John's Wort (*Hypericum perforatum* L.) extract supplementation into diets of laying hens at different levels on performance, egg quality and some blood parameters. International Journal of Science Letters, 2(1), 26–38.

[vms31295-bib-0021] Leke, J. R. , Mandey, J. S. , Ratulangi, F. , Rembet, G. D. , & Junus, C. S. (2018). The effect of Flavonoid papaya seed (*Carica papaya* L) in the organic feed on egg quality and egg shell of local chickens. Journal of Nutrition & Food Sciences, 8(2018), 1–9. 10.4172/2155-9600-C1-053

[vms31295-bib-0022] Li, Y. M. , Su, N. , Yang, H. Q. , Bai, X. P. , Zhu, Q. X. , Liu, H. X. , & Li, J. Q. (2015). The extraction and properties of *Carica papaya* seed oil. Advance Journal of Food Science and Technology, 7(10), 773–779.

[vms31295-bib-0023] Mabusela, S. , Nkukwana, T. , Mokoma, M. , & Muchenje, V. (2018). Layer performance, fatty acid profile and the quality of eggs from hens supplemented with *Moringa oleifera* whole seed meal. South African Journal of Animal Science, 48(2), 234–243.

[vms31295-bib-0024] Maisarah, A. , Asmah, R. , & Fauziah, O. (2014). Proximate analysis, antioxidant and anti proliferative activities of different parts of *Carica papaya* . Journal of Tissue Science and Engineering, 5(1), 1–11.

[vms31295-bib-0025] Meluzzi, A. , Giordani, G. , & Franchini, A. (1992). Influence of breeding technique on some blood parameters of laying hens. Zootec Nutr Anim, 10, 481–488.

[vms31295-bib-0026] Muazu, U. , & Aliyu‐Paiko, M. (2020). Evaluating the potentials of *Carica papaya* seed as phytobiotic to improve feed efficiency, growth performance and serum biochemical parameters in broiler chickens. IOSR Journal of Biotechnology and Biochemistry, 6(1), 8–18.

[vms31295-bib-0027] Nakasone, Y. , & Paull, R. E. (1998). Tropical fruits (Vol. 5). CAB international.

[vms31295-bib-0028] Nkukwana, T. T. , Muchenje, V. , & Masika, P. J. (2014). Proximate composition of pumpkin (*Cucurbita maxima*) seed flour. Basic Research Journal of Agricultural Science and Review, 3(4), 33–35.

[vms31295-bib-0029] North, M. O. (1984). Commercial chicken production manual (3rd ed.). AVI Publ. Comp. Inc.

[vms31295-bib-0030] Novitasari, D. , Mada, U. , Arifah, F. , Mada, U. , Murwanti, R. , & Mada, U. (2018). Estrogenic activity of ethanolic extract of papaya peels (*Carica papaya* L.) on uterine weight and mammae gland proliferation on ovariectomy rats. Indonesian Journal of Cancer Chemoprevention, 9(2), 86–91.

[vms31295-bib-0031] Rahmasari, R. , Hertamawati, R. , Ningsih, N. , Imam, S. , Suryadi, U. , & Nugraha, B. (2022). *Carica papaya* seed meal in diet can reduce egg quail cholesterol without reduce egg quality. IOP Conference Series: Earth and Environmental Science, 980(1), 1–4.

[vms31295-bib-0032] Reece, W. O. , Erickson, H. H. , Goff, J. P. , & Uemura, E. E. (2015). Dukes' physiology of domestic animals. In W. O. Reece (Ed.), Body fluids and homeostasis (13th ed., pp. 114–154). John Wiley & Sons, Inc.

[vms31295-bib-0033] Saleh, A. , Ahmed, E. , & Ebeid, T. (2019). The impact of phytoestrogen source supplementation on reproductive performance, plasma profile, yolk fatty acids and antioxidative status in aged laying hens. Reproduction in Domestic Animals, 54(6), 846–854. 30916364 10.1111/rda.13432

[vms31295-bib-0034] Salla, S. , Sunkara, R. , Ogutu, S. , Walker, L. , & Verghese, M. (2016). Antioxidant activity of papaya seed extracts against H_2_O_2_ induced oxidative stress in HepG2 cells. LWT Food Science and Technology, 66(1), 293–297.

[vms31295-bib-0035] Sanna, M. , Gilardi, G. , Gullino, M. , & Mezzalama, M. (2022). Evaluation of physical and chemical disinfection methods of *Brassica oleracea* seeds naturally contaminated with *Xanthomonas campestris* . Journal of Plant Diseases and Protection, 129(5), 1145–1152.

[vms31295-bib-0036] Soedji, K. , Teteh, A. , Gbeassor, M. , Tona, K. , Nideou, D. , & Decuypere, E. (2017). Effect of *Carica papaya* seeds on gastro‐intestinal parasites of pullet and production parameters. International Journal of Probiotics and Prebiotics, 12(2), 90–96.

[vms31295-bib-0037] Tamiru, B. , Alkhtib, A. , Tamiru, M. , Demeke, S. , Burton, E. , Tolemariam, T. , Debela, L. , & Janssens, G. (2021). Evaluation of dried papaya pomace meal in laying hen diets. Veterinary Medicine and Science, 7(5), 1914–1920. 33955695 10.1002/vms3.516 PMC8464233

[vms31295-bib-0038] Udoyong, A. O , Kibon, A. , Yahaya, S. M. , Yakubu, B. , Augustine, C. , & Isaac, L. (2010). Hematological responses and serum biochemistry of broiler chicken fed graded levels of enzyme (Maxigrain) supplemented cassava peel meal based diets. Global Journal of Biotechnology and Biochemistry, 5(2), 116–119.

[vms31295-bib-0040] Yonatan, K. , Berhan, T. , Etalem, T. , Tesfaye, S. , & Yoseph, C. (2017). Effects of blends of phytobiotic additives of *Nigella satval* L., *Trigonella foenum‐graecum* L. and *Curcuma longa* on serum biochemical profile of broiler chicks. Global Veterinaria, 19(2), 525–531.

